# Dynamic gene expression in fish muscle during recovery growth induced by a fasting-refeeding schedule

**DOI:** 10.1186/1471-2164-8-438

**Published:** 2007-11-28

**Authors:** Pierre-Yves Rescan, Jerome Montfort, Cécile Rallière, Aurélie Le Cam, Diane Esquerré, Karine Hugot

**Affiliations:** 1 National Institute for Agricultural Research, Joint Research Unit for Fish Physiology, Biodiversity and the Environment, INRA Scribe, IFR140, Campus de Beaulieu, 35042 Rennes, France; 2 CRB GADIE, INRA, DGA, Laboratoire de Radiobiologie et d'Etude du Genome, 78352 Jouy-en-Josas, France

## Abstract

**Background:**

Recovery growth is a phase of rapid growth that is triggered by adequate refeeding of animals following a period of weight loss caused by starvation. In this study, to obtain more information on the system-wide integration of recovery growth in muscle, we undertook a time-course analysis of transcript expression in trout subjected to a food deprivation-refeeding sequence. For this purpose complex targets produced from muscle of trout fasted for one month and from muscle of trout fasted for one month and then refed for 4, 7, 11 and 36 days were hybridized to cDNA microarrays containing 9023 clones.

**Results:**

Significance analysis of microarrays (SAM) and temporal expression profiling led to the segregation of differentially expressed genes into four major clusters. One cluster comprising 1020 genes with high expression in muscle from fasted animals included a large set of genes involved in protein catabolism. A second cluster that included approximately 550 genes with transient induction 4 to 11 days post-refeeding was dominated by genes involved in transcription, ribosomal biogenesis, translation, chaperone activity, mitochondrial production of ATP and cell division. A third cluster that contained 480 genes that were up-regulated 7 to 36 days post-refeeding was enriched with genes involved in reticulum and Golgi dynamics and with genes indicative of myofiber and muscle remodelling such as genes encoding sarcomeric proteins and matrix compounds. Finally, a fourth cluster of 200 genes overexpressed only in 36-day refed trout muscle contained genes with function in carbohydrate metabolism and lipid biosynthesis. Remarkably, among the genes induced were several transcriptional regulators which might be important for the gene-specific transcriptional adaptations that underlie muscle recovery.

**Conclusion:**

Our study is the first demonstration of a coordinated expression of functionally related genes during muscle recovery growth. Furthermore, the generation of a useful database of novel genes associated with muscle recovery growth will allow further investigations on particular genes, pathways or cellular process involved in muscle growth and regeneration.

## Background

Food restriction is associated with reduced growth rates. If refed, various animals including fish, grow at a faster than normal rate. During this burst of growth which mainly affects muscle, an accelerated turnover takes place which is characterized by markedly increased protein synthesis relative to degradation [1]. The elevation of protein synthesis after feeding can be translated in terms of the cellular dynamics of muscle growth. Thus, it has been shown that feeding stimulates proliferation of fish myogenic cells *in vivo *[2] as well as *in vitro *[3], providing a source of nuclei for myotube formation and fibre hypertrophy [4]. There is now evidence that muscle recovery growth results from processes of metabolic adaptation, regulated by endocrine as well as the autocrine/paracrine system notably involving IGF1 [1,5,6]. With the purpose of deciphering the mechanisms involved in muscle recovery growth, some studies have also reported the expression of candidate genes such as metabolic-related genes [7], dominant negative regulators of the basic helix-loop-helix (bHLH) transcription factor genes [8] and uncoupling protein 2 genes [9] during nutritional restriction and refeeding in rainbow trout. However, until now the genetic network which is mobilized in recovering muscle has not been exhaustively described. In this study we took advantage of high density trout cDNA microarrays to assess overall gene expression and to determine which pathways are dynamically activated in recovering muscle. Also we identified several genes potentially involved in the gene-specific transcriptional adaptations taking place in recovering muscle.

## Results

### Effect of refeeding on growth characteristics

The mean body weight of the trout was 132 g ± 6.0 and the condition factor was 1.6 ± 0.03 before fasting. At the end of the 30-days fasting period the mean body weight decreased to 121 g ± 5.5 and the condition factor to 1.3 ± 0.03. The mean body weight increased to 130 ± 6.3, 144 ± 7.8, 143 ± 6.7 and 183 g ± 14 and the condition factor to 1.4 ± 0.02, 1.5 ± 0.05, 1.5 ± 0.03, 1.6 ± 0.02, 4, 7, 11 and 36 days post refeeding respectively.

### Changes in gene expression during a fasting-refeeding schedule: Overview

**Figure 1:**
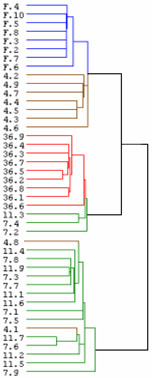
Unsupervised hierarchical clustering consistently sorts fish muscle samples according to feeding conditions. F_2–10_: muscle of distinct fasted trout, 4_1–9_, 7_1–9_, 11_1–8 _and 36_1–9_: muscle of 4, 7, 11 and 36 days distinct refed trout.

**Figure 2:**
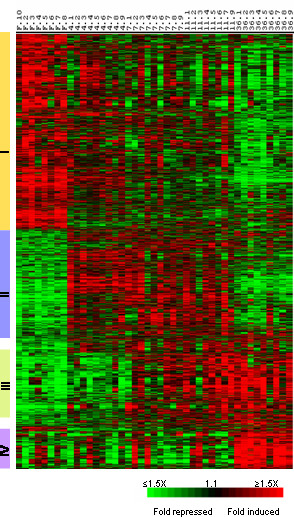
Supervised clustering analysis of the differentially expressed genes selected by SAM. Cluster I comprises genes up-regulated in muscle from fasted animals, cluster II includes genes with transient induction 4 to 11 days post-refeeding, cluster III contains genes whose expression began at 7 days post refeeding and was maintained up to 36 days post-refeeding and cluster IV contains genes up-regulated 36 days post-refeeding. Each row represents the temporal expression pattern of a single gene and each column corresponds to a single sample: columns 1 to 8: muscle from distinct fasted trout ; columns 9 to 17, 18 to 26, 27 to 34 and 35 to 43 : muscle from 4, 7, 11 and 36 day distinct refed trout, respectively. Expression levels are represented by a color tag, with red representing the highest levels and green the lowest levels of expression.

To screen for genes involved in muscle recovery growth, we undertook a time-course analysis of transcript expression in muscle of trout fasted for one month and then refed for 4, 7, 11 and 36 days. At each time point, eight to nine fish were sampled giving in total 43 separate complex cDNA targets that were hybridized to 43 microarrays (GEO accession number: GSE6841). Unsupervised hierarchical clustering of gene expression patterns from all samples produced a consistent grouping of the samples according to the fish feeding conditions (i.e. fasting and 4, 7, 11 and 36 days post-refeeding) (Fig 1). This validated the experimental design and allowed further analysis. To define those genes whose expression levels were significantly different in muscle from 4, 7, 11 or 36 days refed animals compared to muscle from fasted fish we used SAM analysis [10]. We therefore obtained approximately 2200 genes that were then hierarchically clustered using an average-linkage clustering [11]. This resulted in the formation of four major clusters of genes displaying distinct temporal profiles (Fig. 2). A similar clustering was obtained when using the K-means clustering (not shown). The first cluster was composed of genes with peak expression in muscle from starved fish, the second included genes overexpressed at 4, 7 and 11 days post-refeeding and down-regulated at 36 days post-refeeding (cluster II) the third was composed of genes with a later and more sustained induction (7–36 days post-refeeding) (cluster III) and the fourth (cluster IV) contained genes overexpressed only in 36 day refed trout muscle. These expression profiles and the clusters are available online as a browseable file [12]. The accession number of each spotted clones can be obtained by typing the corresponding uniq_id (clone name) in the nucleotide data base of the NCBI.

### Genes with peak expression in starved trout muscle (cluster I)

**Figure 3:**
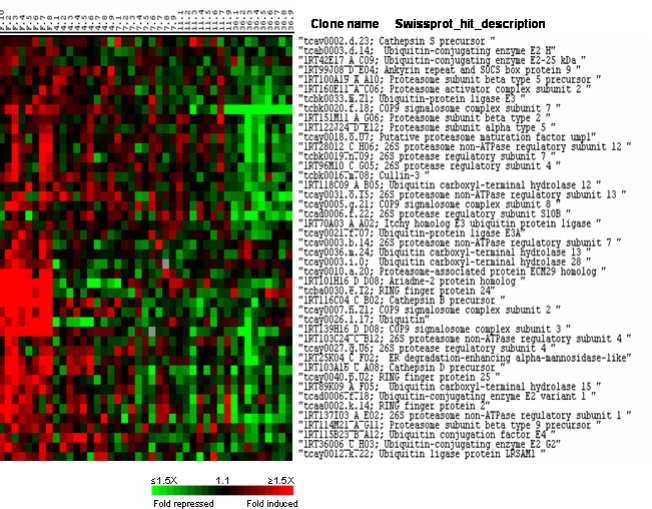
Supervised clustering of SAM selected genes belonging to cluster I and involved in protein degradation. Columns as in figure 2.

Cluster I contained approximately 1000 genes showing high expression in the muscle of fasted fish and down-regulation after refeeding. In this cluster were notably identified two major markers of nutrient deprivation: the tuberous sclerosis component 2 (TSC2) an inhibitor of mTOR function and the translational repressor 4E-BP1. The most distinctive feature of cluster I was the presence of a large repertoire of genes involved in the regulation of protein degradation (Fig. 3). These genes participate either in the lysosomal system such as the cysteine protease cathepsins B, D and S or to the ubiquitin-proteasome pathway. In this latter class were several proteasome subunits, proteasome-associated proteins, ubiquitin, several ubiquitin-conjugating enzymes, ubiquitin carboxyl-terminal hydrolases and ubiquitin ligases. Among other genes involved in proteasome-mediated degradation were several COP9 signalosome complex subunits, cullin-3 and the Ariadne-2 protein homolog. Notably we did not observe any significant up-regulation of calpains in the muscle of fasted trout. Cluster I contained many genes involved in catabolic pathways and beta oxidation of fatty acids such as the short, medium and long chains of Acyl-CoA dehydrogenase.

### Genes up-regulated 4 to 11 days after refeeding (cluster II)

**Figure 4:**
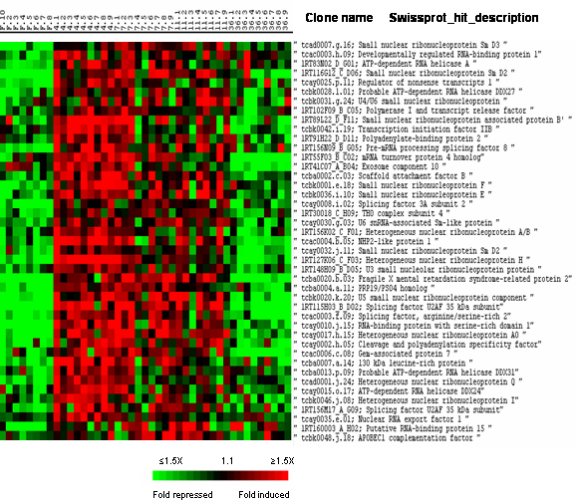
Supervised clustering of SAM selected genes belonging to cluster II and involved in RNA synthesis and processing. Columns as in figure 2.

Cluster II included approximately 550 genes with transient induction 4 to 11 days post refeeding. In this cluster were found more than 40 genes regulating mRNA synthesis, processing and turnover (Fig. 4). Among them were genes encoding small nuclear ribonucleoproteins, transcription initiation factors, RNA helicases of the DDX family, spliceosome-associated proteins such as the NHP2-like protein 1 as well as polyadenylation and export factors.

**Figure 5:**
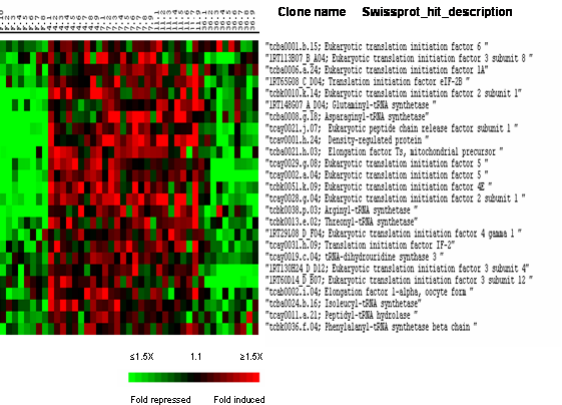
Supervised clustering of SAM selected genes belonging to cluster II and involved in translation. Columns as in figure 2.

**Figure 6:**
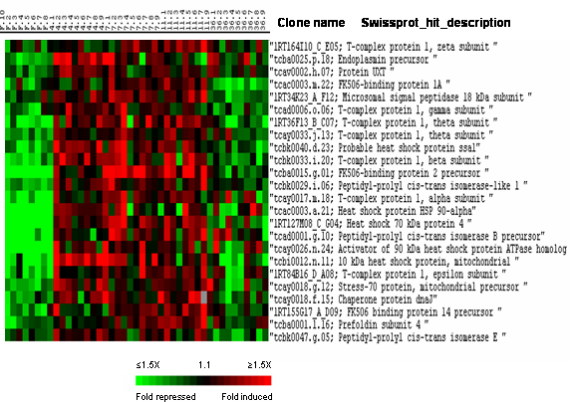
Supervised clustering of SAM selected genes belonging to cluster II and involved in chaperon activity. Columns as in figure 2.

Cluster II also comprised a large set of genes involved in various aspects of translation (Fig. 5). These genes encode translation initiation factors (including the SUI1 domain containing density-regulated protein DRP), elongation and peptide chain release factors, peptidyl-tRNA hydrolases and several aminoacyl-tRNA synthases. In addition to these genes, a large group (Fig. 6) of genes whose products regulate protein folding and maturation was also found in cluster II. Among these latter were several heat shock proteins (HSP), endoplasmin, several subunits (alpha, beta, gamma, theta, zeta and epsilon) of the chaperonin-containing complex TCP1 and seven peptidyl prolyl cis/trans-isomerases encoding genes (including genes encoding FK506-binding proteins) that are known to catalyze the *cis-trans *isomerization of prolyl bonds in oligopeptides and various folding states of proteins

**Figure 7:**
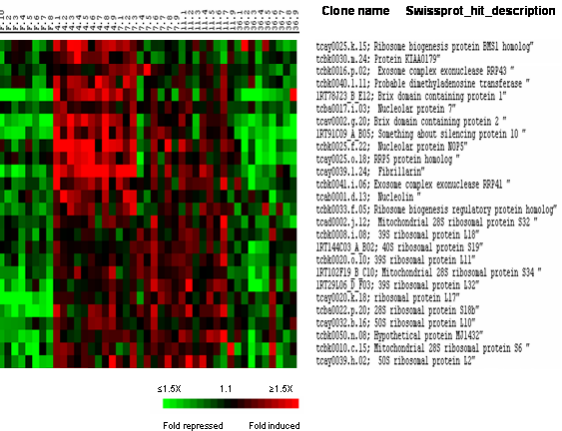
Supervised clustering of SAM selected genes belonging to cluster II and involved in ribosome formation. Genes regulating ribosome biogenesis segregate from genes encoding ribosomal proteins forming a subcluster peaking at 4 days post-refeeding. Columns as in figure 2.

Consistent with the induction of genes involved in protein biosynthesis, cluster II included a large number of genes involved in ribosome formation. This group of genes which is presented in Fig. 7 included structural genes encoding ribosomal proteins as well as several genes whose products regulate ribosome biogenesis among which were fibrillarin, nucleolin, Brix domain-containing proteins, PUA domain-containing hypothetical protein MJ1432, RRp5, BMS1 and SAS 10 (something about silencing protein 10). Interestingly, in contrast to ribosomal structural genes which displayed about uniform expression during the 4 to 11 days post refeeding period, most of the genes regulating ribosome biogenesis sub-clustered together (Fig. 7, upper part) peaking at 4 days post-refeeding.

**Figure 8:**
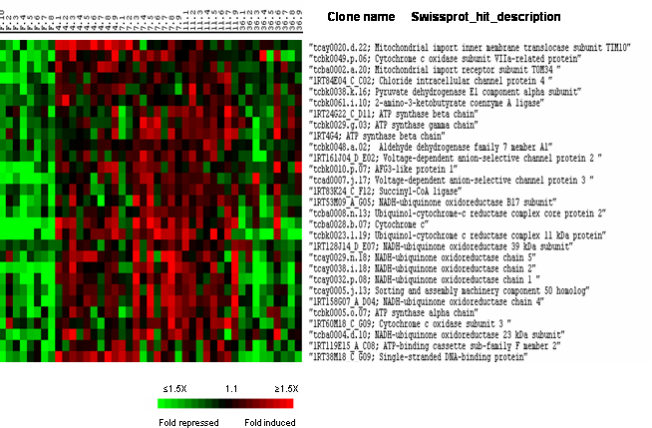
Supervised clustering of SAM selected genes belonging to cluster II and involved in mitochondrion biogenesis and activity. Columns as in figure 2.

In accordance with an increase in cellular biosynthesis, a large number of genes involved in mitochondrial production of ATP grouped into cluster II (Fig. 8). Among them were several genes which are components of the oxidative phosphorylation system (chains 1, 2, 4 and 5 of the NADH-ubiquinone oxidoreductase, cytochrome c and ubiquinol-cytochrome c reductase complex subunits) as well as several genes of the ATP synthase complexes (alpha, beta and gamma chains). Along with these genes involved in energy production, we observed the induction of several genes important for mitochondrion formation or biogenesis such as TIM10 (a mitochondrial import inner membrane translocase), TOM34 (a mitochondrial import receptor subunit), voltage-dependant anion-selective channel protein 2 and 3 and the metalloprotease AFG3-like protein 1. Some enzymes of the mitochondrial matrix such as pyruvate dehydrogenase isoforms and succinyl-CoA ligase were also found in this cluster. Consistent with a stimulation of mitochondrial biogenesis the gene encoding the mitochondrial single-stranded DNA-binding protein (Mt-SSB) involved in mitochondrial DNA replication was also up-regulated.

**Figure 9:**
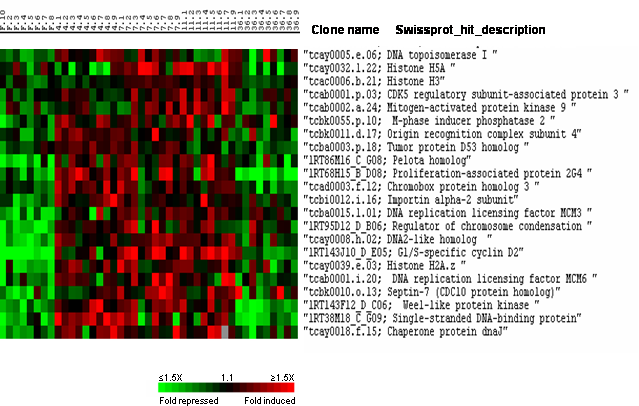
Supervised clustering of SAM selected genes belonging to cluster II and involved in cell division and chromatin assembly. Columns as in figure 2.

Cluster II also contained several genes characteristically expressed during cell proliferation (Fig. 9). These genes are involved in DNA replication (mcm3, mcm6, DNA2-like homolog, DNA topoisomerase I and origin recognition complex subunit 4), progression through the cell cycle (G1/S-specific cyclin D2, CDK5 regulatory subunit, Wee1-like protein kinase, mitogen-activated protein kinase 9, M-phase induced phosphatase 2, pelota homolog and tumor protein D53 homolog), chromosome condensation (histone H2Az, H3, H5A, chromobox protein homolog 3 and regulator of chromosome condensation) or associated with proliferation (protein 2G4).

Among the most differentially regulated genes with miscellaneous functions and belonging to cluster II, we found uridine-cytidine kinase 2, various genes preventing cell apoptosis such as MCL1. Genes whose products mediate cAMP-dependant signalling such as the cAMP-dependent protein kinase beta catalytic subunit were also present. Among the genes belonging to cluster II with unknown functions we found C9orf32, the surfeit locus protein 2 and 4 encoding genes, the genes for the hypothetical protein ZK637.2 and for the hypothetical WD-repeat protein C1A6.02 in chromosome I.

### Genes up-regulated 7–36 days after refeeding (cluster III)

**Figure 10:**
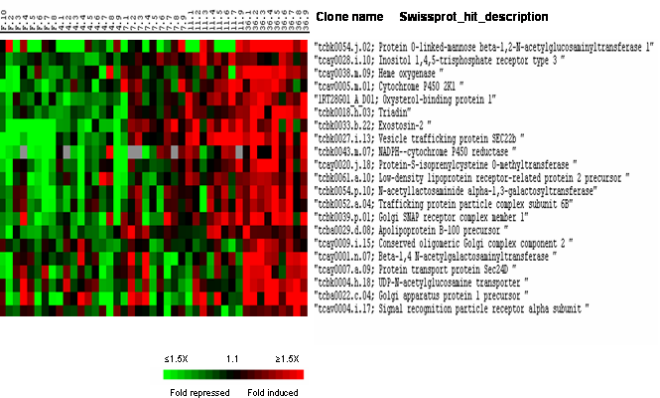
Supervised clustering of SAM selected genes belonging to cluster III and IV and involved in reticulum and Golgi dynamics. Columns as in figure 2.

**Figure 11:**
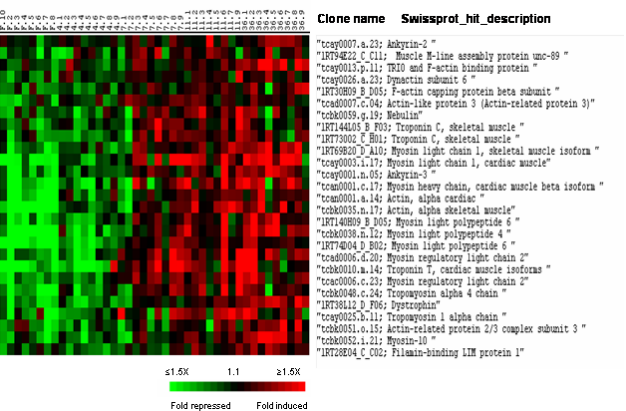
Supervised clustering of SAM selected genes belonging to cluster III and involved in cytoskeletal and myofibrillar organisation. Columns as in figure 2.

Cluster III that contained approximately 480 genes up-regulated 7 to 36 days post refeeding was enriched in genes that encode components of the reticulum and Golgi apparatus such as triadin, and exostosin-2, and proteins involved in transport from the reticulum to the Golgi apparatus including golgi SNAP receptor complex member 1 (Fig. 10). Numerous genes in cluster III function in actin cytoskeletal rearrangements (including dynactin subunit 6, actin-like protein 3, actin-related protein 2/3 complex subunits and ankyrins) and organisation of the sarcomere (skeletal actin, myosins, tropomyosins, troponins, nebulin) (Fig. 11). Also were grouped in cluster III several collagen genes (collagen alpha 1(I), alpha 2(I), alpha 5 (IV) and alpha 1 (V) chains) that participate in the synthesis of the muscle extracellular matrix. Among the genes of cluster III with miscellaneous function were several Ras-related proteins such as Rab-24, Rab-26, and Rab-11b, several members of the glutathione S-transferase family, the bifunctional methylene tetrahydrofolate precursor and the myeloid leukaemia factor 1 (MLF1).

### Genes up-regulated in the muscle of 36 days refed trout (cluster IV)

Cluster IV contained fewer (less than 200) genes compared to the other clusters. As observed for cluster III, cluster IV comprised several genes regulating reticulum and Golgi biogenesis and activity such as glycosyltransferases and the protein transport Sec24D (Fig. 10). A distinctive feature of cluster IV was to contain several genes encoding glycolytic enzymes (triose-phosphate isomerase, 6-phosphofructokinase, alpha-enolase and phosphoglycerate kinase). In addition, cluster IV contained several genes involved in lipid biosynthesis such as 24-dehydrocholesterol reductase precursor, ethanolamine kinase, Scavenger receptor class B member 1 and 1-AGP acyltransferase.

### Functional categorization of genes contained in clusters I–IV as shown by GoMiner algorithm

**Figure 12:**
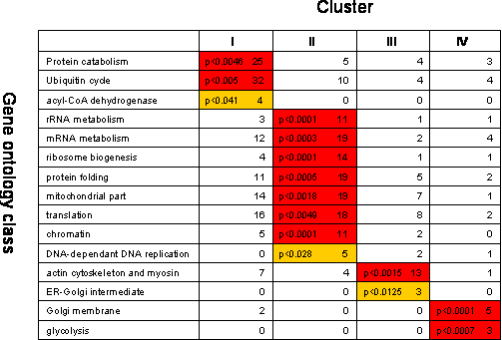
Ontology-matrix showing functional grouping of genes belonging to clusters I–IV. The enrichment of genes of a given functional class is indicated for each cluster. Also are represented the p-values. Dark and light colors represent P-values of < 0.005 and < 0.05 respectively

To further assess the enrichment of a particular functional class of genes in each cluster we used the GoMiner algorithm [13]. Given that the GO data bases that served as input for GoMiner did not encompass the whole of genes present in our membrane only a subset of genes contained in cluster I–IV was selected for GoMiner analysis. Nevertheless, in agreement with the detailed identification of the genes contained in the different clusters (see above), the GoMiner notably highlighted an enrichment of cluster I for genes regulating protein degradation while cluster II was found to be enriched for genes involved in RNA metabolism (and more particularly rRNA metabolism), ribosome biogenesis, mitochondrion, translation and protein folding (Fig. 12). On the other hand, cluster III was particularly found to be enriched for genes participating in actin cytoskeleton and myofibrillar organisation (Fig. 12). This functional categorization was supported by probability values (Fig. 12) which yielded the measure of the likelihood that a particular biological process was overrepresented in a cluster compared with that expected by random selection from the SAM list.

### Transcriptional regulators induced during muscle recovery growth

**Figure 13:**
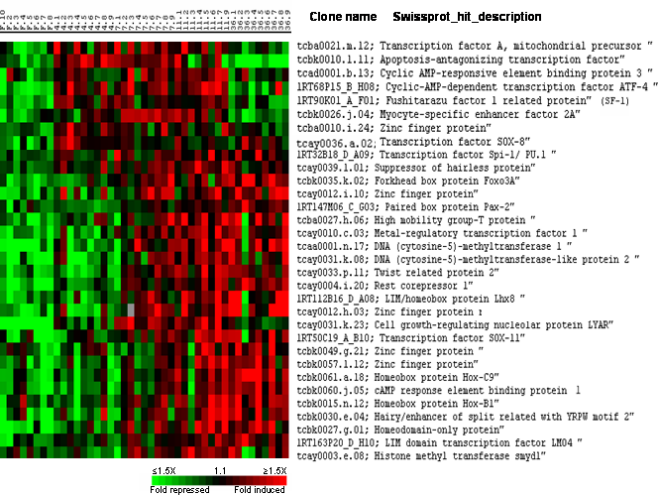
Supervised clustering of SAM selected genes induced during muscle recovery growth and involved in transcriptional regulation. Columns as in figure 2.

Some of the genes induced during muscle recovery growth were themselves regulators of transcription (Fig. 13). Among those present in cluster II, were two cyclic AMP-dependent transcription factors closely related to CREB3 and to ATF4 respectively, MEF2a, SF-1/fushitarazu homolog 1 related protein, cell growth regulating nucleolar protein LYAR, apoptosis-antagonizing transcription factor AATF and an unidentified zinc finger protein encoding gene. Cluster III included MTF1, the LIM/homeobox protein Lhx8, the homeobox proteins Hox-C9 and Hox-B1, the LIM domain containing transcription factor LMO4, the Homeodomain only protein Hop, a CREB1 related protein, sox11 as well as four unidentified zinc finger protein encoding genes. In addition to transcriptional regulators that trigger gene-specific expression by binding to sequence of promoters, we found other genes overexpressed during muscle recovery growth which potentially control gene expression by inducing histone modifications (SmyD1) or DNA modification (DNMT1 and DNMT2).

### Validation of the microarray gene expression data

**Figure 14:**
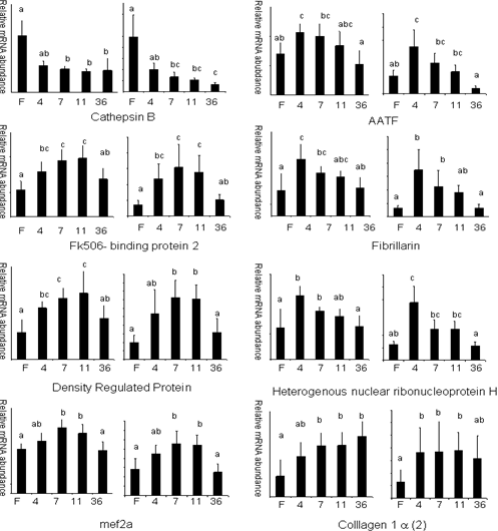
Relative mRNA expression levels of selected genes in muscle from fasted (F), 4 (4), 7 (7), 11 (11) and 36 (36) day-refed trout as obtained by microarray hybridisation (left) and Q-PCR (right). The mRNA levels measured by Q-PCR were normalized to levels of 18S rRNA. Bars sharing the same letter(s) are not significantly different (p < 0.05).

The accuracy and reliability of the results obtained with microarrays were tested by quantitative RT-PCR (Q-PCR) of 10 selected genes belonging to different functional classes. The gene expression levels obtained by Real-time PCR were normalized to that of the 18s. A good agreement was observed between microarray and Q-PCR analysis (Fig. 14) with discordant results obtained for only two genes. Thus, differential expression detected by microarray analysis is highly predictive of expression levels measured with an independent methodology such as Q-PCR (80% confirmation).

## Discussion

In this study we used high density cDNA arrays to describe the changes in gene expression during muscle recovery induced by a fasting-refeeding sequence. Statistical analysis of the microarray data and transcriptional profiling led to the identification of 4 major temporally distinct clusters. The detailed identification of the genes contained in each cluster along with a Gominer analysis showed that these clusters contained genes involved in distinct biological functions.

In agreement with the proteolytic and lypolytic responses to starvation described in mammals [14] a large set of genes overexpressed in the muscle of fasted trout and down-regulated after refeeding, revealed an adaptation program that favours fatty acid oxidation and protein degradation to fuel metabolism. Two pathways of proteolysis appeared to be mobilized in the muscle of fasted trout: the lysosomal system as revealed by the up-regulation of several cathepsins and the ubiquitin-proteasome system, as indicated by the overexpression of numerous components involved in its activity. On the other hand, in agreement with a previous report showing that transcripts of calpains are not changed in muscle proteolysis of gravid trout [15], calpain genes were not found to be up-regulated in fasted trout muscle. Overall our data are consistent with the notion that most of the accelerated proteolysis in muscle is due to an activation of the Ub-proteasome pathway [16]. Supporting an increased protein degradation relative to synthesis due to food deprivation, starved trout muscle contained high levels of the transcript for tuberous sclerosis component 2 (TSC2) which has been shown, in mammals, to inhibit mTOR a positive regulator of cell growth and proliferation [17], and of the transcript encoding the translational repressor 4E-BP1, which presumably reinforces the inhibition of cap-dependent translation resulting from inactivation of Akt/mTOR [17].

Most of the genes that were up-regulated during muscle recovery growth fell into three major clusters (II–IV) with distinct temporal profiles and showed remarkable consistency in their functional categories. Cluster II which corresponds to the first phase of muscle recovery contained a large set of genes that stimulate cellular biosynthesis. This cluster included genes involved in transcript processing, translation or involved in ribosome production. A large number of genes was also found in this cluster that are involved in post-translational modifications of nascent proteins such as heat shock proteins, subunits of the chaperonin containing t-complex polypeptide 1 and peptidyl-prolyl cis trans isomerases that are chaperone enzymes which alter the peptide bond between a given amino acid and a proline, changing it from the *cis *to the *trans *conformation and *vice versa*. Interestingly, it has been previously reported in zebrafish that the heat shock protein hsp90 alpha not only participates in the correct protein folding but also plays a specific role in the normal process of myogenesis [18]. Given that hyperplasia (neosynthesis of myofibres) contributes to muscle growth in fish [19], it can be speculated that an elevated level of hsp 90 alpha in recovering muscle is at least in part related to the differentiation of neomyofibres. Several genes up-regulated during muscle recovery encode protein regulating the cell cycle and mitosis. This suggests, in agreement with a previous work carried out in the Antarctic fish *Notothenia coriiceps *[2] that cell proliferation is stimulated by feeding, increasing myoblast fusion into new myotubes (hyperplasia) and providing a source of nuclei as muscle fibres increase in diameter (hypertrophy). Consistent with cell cycle progression and cellular growth, which require adjustment in mitochondrial ATP production, cluster II was found to contain several transcripts that encode proteins required for oxidative respiration and ATP synthesis or involved in mitochondrial biogenesis.

Cluster III contained genes associated with Golgi and reticulum dynamics. The induction of these genes, most of which participate in post-translational modifications and transport of proteins, is consistent with the enhancement, 4 to 11 days post refeeding, of the protein synthesis machinery. A striking feature of cluster III is the presence of a large set of genes encoding cytoskeletal proteins and sarcomeric proteins involved in contractile functions. At the same time are induced several transcripts encoding matrix compounds such as chains of the fibril forming collagen I which is the major collagen of intramuscular connective tissue in fish [20]. All these expressions that evoke the muscle regenerative response that follows cardiotoxin delivery in mouse [21] show that myofiber and muscle remodelling occur at a late time period of muscle recovery growth. As in cluster III, cluster IV contained genes regulating Golgi and reticulum biogenesis and activity but differed by the presence of genes with a role in carbohydrate metabolism and lipid biosynthesis. The increased expression in recovering trout muscle of genes involved in glycolysis is in agreement with recent data showing that atrophying muscle of gravid trout has low levels of transcripts encoding many glycolytic enzymes [15].

Among the transcriptional regulators induced in recovering muscle was found MEF2a. The transcription factors of the MEF2 family bind to an A/T rich sequence present in many muscle-specific promoters and enhancers [22]. Supporting a role of MEF2a in transcription of fish contractile protein encoding genes, MEF2 sites have been identified in carp myosin promoters [23] and MEF2a knockdown in zebrafish has been shown to induce a down regulation of a large set of transcripts for gene encoding contractile proteins such as troponins, myosin heavy and light chains and α-tropomyosin [24]. Interestingly MEF2a induction in recovering muscle precedes that of the sarcomeric protein encoding genes. This temporal sequence is in accordance with a function of MEF2a in the burst of myofibrillar protein encoding genes activation observed during muscle compensatory growth. Also were induced Sox8, Sox11 and the LIM-only protein gene LMO4. All these genes are expressed in the fish differentiating embryonic myotome [[25] and [26]; Dumont and Rescan: unpublished results] suggesting that their expression in recovering muscle relates to the formation of new differentiating muscle fibres. Also the induction of Hop (Homeodomain only protein) in trout recovering muscle is of interest because Hop has been recently reported in mammals to regulate skeletal myoblast differentiation and to play a critical role in muscle regeneration [27]. The induction of three cyclic AMP-responsive element binding proteins (ATF4, CREB1 and CREB3) along with cAMP-dependent protein kinase subunits suggests a possible role of cyclic AMP signalling in the recruitment of new myofibres during muscle recovery. Supporting this view, it has been reported in amniotes that the cAMP pathway participates in the regulation of myogenesis [28] involving a CREB-mediated transcription [29] and that ATF4 is induced during *in vitro *differentiation of human skeletal myoblasts [30]. In addition to the induction of transcriptional regulators that exert their function at the nuclear genome level, we observed the up-regulation of the transcription factor A that is a key activator of mitochondrial transcription and a participant in mitochondrial genome replication [31] Several other genes up-regulated during muscle recovery growth fall within the transcriptional regulator class. These genes either have homology to known sequences, but no demonstrated function in muscle development and growth (for example: LYARR, the leucine zipper containing factor AATF and PU.1) or have no homology to any known sequence (in particular most of the zinc finger protein genes). All these genes deserve in-depth studies: it would be of interest in particular to identify their individual function in fish muscle development and growth using antisens morpholino oligonucleotides and to further characterize their gene targets. In addition to the up-regulation of transcription factors, some genes related to chromatin remodelling or DNA modification also were found to be induced in recovering muscle. Thus we observed the induction of the chromatin remodelling protein SmyD1 and that of two DNA (cytosine-5) methyl transferases (DNMT1 and DNMT2). SmyD1 has been recently shown to have a major role in myofibril organisation in the zebrafish embryo [32]. Consistent with such a function in recovering trout muscle it is interesting to note that SmyD1 is coexpressed with genes encoding sarcomeric proteins. In relation to a function for DNMT1 in muscle growth, it has been reported that forced expression of DNMT1 in murine C2C12 myoblasts causing *de novo *methylation in the MyoD gene induced its expression and stimulated myogenesis [33].

## Conclusion

Our microarray analysis shows that genes overexpressed in recovering muscle fall into distinct groups with distinct temporal profiles which showed remarkable consistency in their functional classes. The early phase of muscle recovery was associated with dramatic transient induction of a large number of genes functionally related to RNA processing, translation, maturation of proteins, ribosome biogenesis, cell proliferation and mitochondrial bioenergetics. In a later and more sustained phase several genes regulating Golgi and reticulum dynamics and genes involved in muscle remodelling were induced. The generation of a database of novel genes associated with muscle recovery growth will help investigations on genes, pathways or cellular process involved in muscle growth and regeneration.

## Methods

### Animals and experimental design

Investigations were conducted in agreement with the guiding principles for the use and care of laboratory animals and in compliance with European regulations on animal welfare. A spring strain of l-year-old rainbow trout (*Oncorhynchus mykiss*) was used. The fish had been fed to satiation with a commercial diet until the beginning of the starvation. The fasted group was composed of fish initially weighing about 130 grams that were deprived of food for 30 days. Refed groups were composed of trout from the fasted group that were fed at a rate three time higher than the normal ration and sampled sequentially at 4, 7, 11 and 36 days post refeeding. Fish were reared in freshwater tank (PEIMA-INRA, Sizun, France) under a natural photoperiod. The water temperature was 11.8°C at the end of starvation, 11.1°C, 10.5°C, 10.4°C and 7.8°C, 4, 7, 11 and 36 days post refeeding respectively. The fish were rapidly anaesthetized with phenoxy-ethanol (Aquaveto, 4 ml per 10 liters of fresh water) before dissection. The condition factor (an indicator of the body shape) was calculated as follows: K = body weightx100/body length ^3 ^(the body length did not include the caudal fin length).

### RNA purification and complex cDNA target preparation

Muscle from 8 or 9 trout was sampled for each time point. A transverse slice of fast muscle situated just beneath the dorsal fin was taken for RNA extraction. Total RNA was purified using TRIzol reagent (Invitrogen, Carlsbad, CA). The RNA integrity and concentration were respectively controlled and calculated with the Agilent bioanalyser. Complex target were prepared from 5 μg of RNA of each sample by simultaneous reverse transcription (using oligo(dT) as primer) and labelling for 2 hours at 42°C in the presence of 30 μCi [alpha-33P] dCTP, 120 μM dCTP, 20 mM each dATP, dTTP, dGTP and 400 units Superscript II reverse transcriptase (Invitrogen). RNA was degraded by treatment at 68°C for 30 min with l μl 10% SDS, l μl 0.5 M EDTA and 3 μl 3 M NaOH, and then equilibrated at room temperature for l5 min. Neutralization was done by adding 10 μl 1 M Tris-HCI plus 3 μl 2 N HCl.

### cDNA microarrays production

Nylon micro-arrays (7.6 × 2.6 cm) were obtained from the INRA-GADIE resource centre [34]. A set of 9023 distinct rainbow trout cDNA clones originating from pooled-tissues libraries [35,36] were amplified by PCR using primers specific of the polylinker sequence of the vectors. Quality of the amplification products was systematically checked on 1% agarose gels. Unpurified PCR products were evaporated, resuspended in 20 μl of distilled water, then transferred to 384-well microplates and spotted onto nylon membranes (Hybond-N+; Amersham Biosciences, Saclay, France). The last step was conducted using a Biorobotics MicroGrid-II arrayer (Genomics Solution, Cambridge, U.K.) equipped with a 64-pins Biorobotics printhead and 64 Biorobotics 100 μm solid pins. The spotted DNA were denaturated in 150 mM NaOH, 1.5 M NaCl. A neutralisation step was performed in 1 M Tris HCl (pH 7.5), 1.5 M NaCl. A last step to rinse micromembranes in 2 × SSC was performed. The DNA was subsequently fixed by successive heat (80°C during 2 hours) and UV (120000 μJ) treatments.

### Hybridisation

A first hybridization was performed using a 33P-labelled oligonucleotide (TAATACGACTCACTATAGGG) which is found at the extremity of each PCR product to monitor the amount of cDNA present in each spot. After stripping (3 hours 68°C, 0.1 SSC, 0.2% SDS), arrays were prehybridized for 4 h at 65°C in hybridization solution (5× Denhardt's, 5 SSC, 0.5% SDS) and hybridized with denatured labeled cDNAs for 48 h at 65°C in hybridization solution. After 3 washes (1 hour 68°C, 0.1 SSC 0.2% SDS), arrays were exposed for 65 hours to phosphor-imaging plates before scanning using a FUJI BAS 5000. Signal intensities were quantified using BZScan software [37].

### Microarray signal processing

Low oligonucleotide signals (lower than three times the background level) were excluded from the analysis. After this filtering step, signal for each spot was divided by the hybridisation signal obtained with the vector oligonucleotide. After this correction, signals were then normalized by dividing each gene expression value by the median value of the array.

### Microarray data analysis

SAM software [10] was used to identify genes differentially expressed between muscle of fasted trout and muscle of 4, 7, 11 and 36 day refed trout. For each comparison a false discovery rate (FDR) of 0.01% was used. All genes identified in at least one of the above comparisons were kept for clustering analysis in order to characterize the temporal expression profiles of statistically relevant genes. For supervised clustering analysis, data was log transformed, median-centred and an average linkage clustering was carried out using CLUSTER software and the results were visualized by TREEVIEW [11].

### Data mining

Rainbow trout sequences originating from INRA Agenae [35] and USDA [36] EST sequencing programs were used to generate publicly available contigs [38]. The 8th version (Om.8, released January 2006) was used for Blast X comparison against the Swiss-Prot database (January 2006) [39]. The score of each alignment was retrieved after performing a BlastX comparison. In addition, for each EST spotted onto the membrane, the accession number of the corresponding rainbow trout cluster (Unigene Trout, January 2006), if any, was retrieved from the UniGene database [40].

### Gene Ontology analysis

In order to assign functional categories to each identified clusters, we used GoMiner software [13]. For each PCR product spotted, the corresponding contig was blast against Swissprot Database (a score > 86 was considered as significant). Then the SwissProt accession number was used as the input to analyse gene lists of clusters for GO categories that showed statistically enrichment (2265 genes obtained by SAM analysis as total genes list). P-values were estimated by two-sided Fisher's exact test and only P < 0.05 were retained.

### Real-time PCR analysis

The expression of 10 selected genes belonging to different functional classes was analysed by real-time RT-PCR. Total RNA (2 μg) from all samples used for microarray analysis were reverse transcribed using 200 Units of moloney murine Leukemia virus (MMLV) reverse transcriptase (Promega) and 0.5 μg random hexamers (Promega) per μg of total RNA according to manufacturer's instruction. The expression levels were determined using the I-cycler IQ (Biorad, Hercules, CA) instrument. Briefly reverse transcription products were diluted to 1/24 and 5 μl were used for each real-time PCR reaction which were performed using a real-time PCR kit provided with a SYBR Green fluorophore (Eurogentech, Belgium). For each target gene, primers (Table 1) were chosen to presumably flank an intron as determined by comparison with the gene sequence of zebrafish ortholog. The amount of the target RNA was determined by comparison with a standard curve generated using serial dilution of RT reactions. This dilution curve was used to ensure that PCR efficiency ranged from 90 to 100% and that amplification was linear within sample set. The level of 18S RNA in each sample was also measured by real-time RT-PCR and used for target genes abundance normalisation within sample set. Statistical analysis were performed using Statistica 7.0 software (Statsoft, Tulsa, OK). Differences between samples were analysed using non parametric Kruskal-Wallis test.

**Table 1:** Sequence of the primer pairs used for real-time quantitative PCR and gene names.

**Abbreviated name**	**Sequence**	**Target gene**
HNNP-FW	GTAGAGCTGGAGTCGGAGGA	Heterogeneous nuclear ribonucleoprotein H
HNNP-RV	CCTCAGTCTCTGGGCAGTTT	
MEF2A-FW	GCTCAGGCCTCCTTACACAG	Myocyte enhancer factor 2a
MEF2A-RV	ACTTGGTGGGCATGACTTTC	
DRP-FW	AGGTCCGGTAGGAAATGCTT	Density Regulated Protein
DRP-RV	ACGGGGGATTTTAGCTATCG	
FBL-FW	AAACCTGGCTCGAAGGTCAT	Fibrillarin
FBL-RV	TGGTTCTCTTTTTGGCAACG	
FK506bp2-FW	CCTGCTCGGAATGTGTGAG	FK506-binding protein 2
FK506bp2-RV	AAGTCAGACCTCCTCTCGATG	
AATF-FW	CATTCAGAAGCTCCGCAGTA	Apoptosis antagonizing transcription factor
AATF-RV	TCCCGAATAGTGAGCGAAAC	
Cathps-FW	CTCAGACCGCGTGTGTATCC	Cathepsin B
Cathps-RV	GACCAGCCCCTCTTTAGTCC	
Coll I α2-FW	CTCGAGTCTGCGTGGACAT	Collagen I alpha 2 chain
Coll I α 2-RV	GTCCGATAGCACCATGACCT	

## Authors' contributions

PYR coordinated the study, analysed the data and wrote the manuscript. JM and AL performed microarray experiments and participated in the data analysis. CR performed RNA extractions and Real-time PCR. DE and KH prepared the microarrays. All authors read and approved the final manuscript.
